# Osteoid Osteomas of the Talus: A Case Report of Four Patients

**DOI:** 10.7759/cureus.40798

**Published:** 2023-06-22

**Authors:** Mark D Wishman, Jensen Henry, Carson Rider, Carolyn Sofka, Edward Yoon, Andrew Elliott

**Affiliations:** 1 Department of Foot and Ankle Surgery, Hospital for Special Surgery, New York, USA; 2 Department of Foot and Ankle Surgery, Campbell Clinic Orthopaedics, Germantown, USA; 3 Department of Radiology and Imaging, Hospital for Special Surgery, New York, USA

**Keywords:** ankle, hindfoot, radio-frequency ablation, osteoid osteoma, talus

## Abstract

Osteoid osteomas are benign bone tumors that are commonly found in the cortical segments of long bone but can occasionally occur in the talus of the foot. They typically present in younger males and are characterized by lesions with a vascularized nidus surrounded by sclerotic bone. Plain radiographs can often miss the diagnosis, requiring further imaging with computed tomography (CT) or magnetic resonance imaging (MRI). Lesions often lead to a significant inflammatory response resulting in an impaired range of motion and nocturnal pain. Conservative management with non-steroidal anti-inflammatory medications and a walking boot is considered first-line therapy, with failure to respond being an indication for surgical intervention. Surgical treatment traditionally consisted of en bloc resection but has been replaced by CT-guided radio-frequency ablation (RFA) when conservative management has failed. Four cases of osteoid osteoma of the talus are presented which all went on to RFA after conservative management failed. The patients’ non-specific symptomatology and unremarkable findings on plain radiographs led to further evaluation using MRI or CT, which aided in the diagnosis. Following imaging, RFA was performed which resulted in 100% relief of pain and symptoms in all four patients and a return to full activity without limitations. Osteoid osteomas of the talus present unique challenges due to the non-specific symptoms and complex surrounding anatomy that accompanies this condition. Management should include the use of CT for localization and RFA of the lesion, which we have shown leads to complete resolution of symptoms and return to normal daily activities.

## Introduction

Originally characterized by Jaffe in 1935 [1], osteoid osteomas are osteoblastic bone tumors that account for approximately 10% of all benign bone neoplasms [2]. Affected patients are usually younger than 30 and more frequently are males [3]. These tumors are commonly located in the cortical segments of long bones but have also been reported in the spine, pelvis, foot, and ankle [4]. Most osteoid osteomas are isolated small lesions consisting of a highly vascularized nidus surrounded by sclerotic bone with or without calcifications.

Patients with osteoid osteomas often present with edema, decreased range of motion, and most characteristically, nocturnal pain. The severe pain associated with osteoid osteomas has been linked to excess prostaglandin production, reaching levels 30x higher than normal bone. This increase in prostaglandin production from within the nidus leads to extreme inflammation and pain [5,6]. Medical management of this condition using non-steroidal anti-inflammatory drugs (NSAIDs) often provides symptomatic improvement; however, this does not lead to a change in the characteristics or presence of the lesion [7].

The most common finding on plain radiographs is a radiolucent nidus within a zone of sclerotic bone. In certain cases, such as intra-articular osteoid osteomas, the complexity of the surrounding anatomy can conceal the nidus on plain radiographs, revealing only encircling sclerosis. Therefore, the preferred method of diagnosis and localization is via computed tomography (CT) or magnetic resonance imaging (MRI), in which a well-defined nidus, occasionally with calcifications, can be seen surrounded by a region of sclerotic bone [8]. Bone scintigraphy has also been used for precise localization of the nidus and can be seen with a characteristic double-density sign [9].

Surgical treatment of osteoid osteomas historically consisted of en bloc resection or curettage. These techniques allow for the effective removal of the nidus and sclerotic bone, as well as the ability to perform histology to confirm the diagnosis and complete resection. However, the removal of large portions of bone is unfavorable due to increased fracture risk, the need for bone grafting, and longer recovery times. Starting in the 1990s, surgical treatment of osteoid osteomas was accomplished via radio-frequency ablation (RFA) or thermo-coagulation guided by CT. The benefits of these methods include reduced bone loss as well as faster recovery times but can be complicated when complex neurovascular anatomy is situated nearby or when there is a recurrence of osteoid osteomas after primary treatment [10].

Osteoid osteomas of the talus occur in less than 3.4% of cases and present unique challenges due to the complex anatomy of the area including the partial intra-articular nature of the bone [11]. Differential diagnosis includes osteoid osteoma, osteomyelitis with Brodie’s abscess, stress fracture, and osteoblastoma. Initial treatment consists of NSAIDs for pain management, followed by removal of the lesion by RFA. While cure rates are high using this method, occasional complications including neurovascular injury, cutaneous necrosis, and osteonecrosis can occur [12].

## Case presentation

Case one

**Figure 1:**
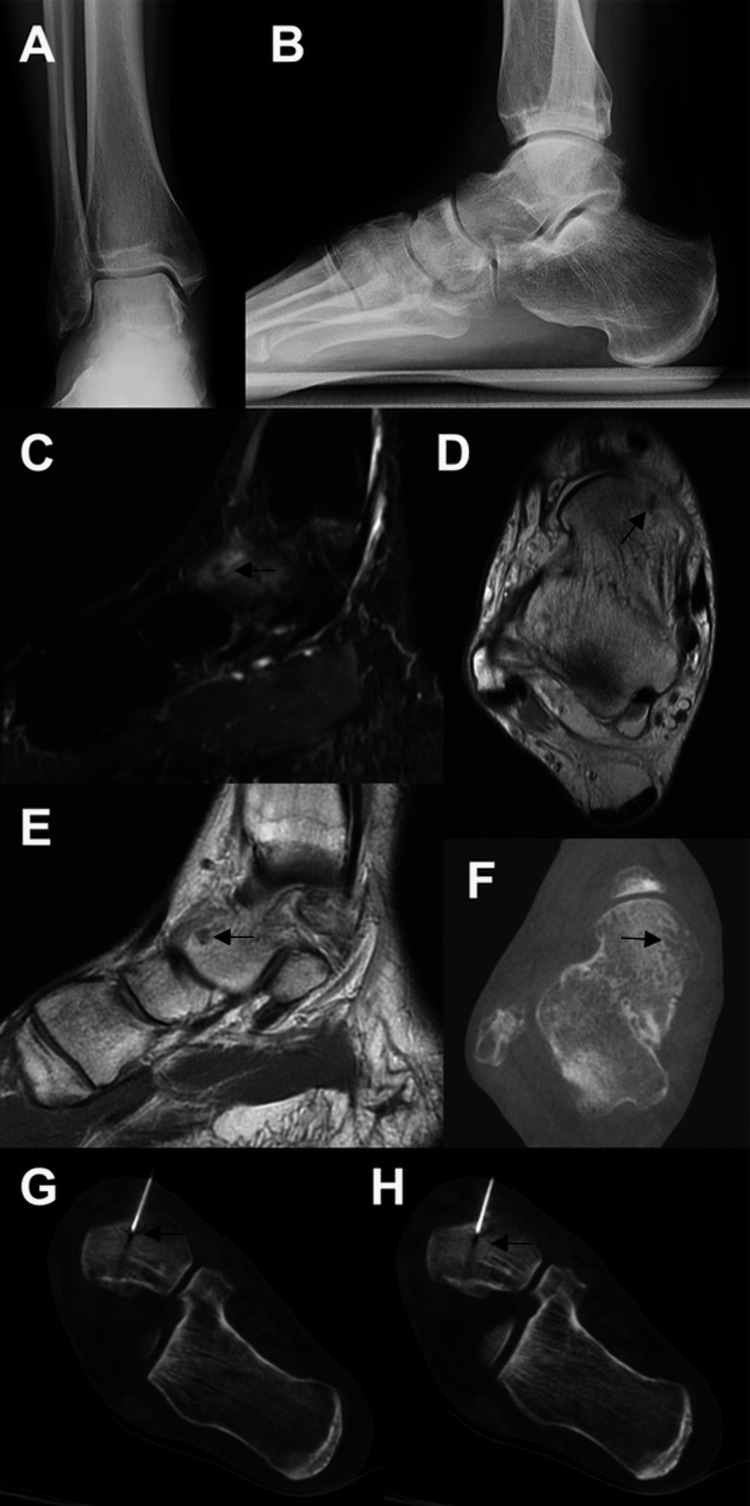
Images of the patient discussed in case one. Initial weight-bearing AP (A) and lateral radiographs (B) were unremarkable. The osteoid osteoma lesion is better delineated on the MRI; sagittal inversion recovery (C), axial proton density (D), and sagittal proton density (E) images are shown. The lesion was also seen on the weight-bearing axial CT (F). Intra-procedure CT images demonstrate successful targeting of the lesion with RFA (G, H). MRI: magnetic resonance imaging; CT: computed tomography; RFA: radio-frequency ablation

A 30-year-old male presented with a one-year history of right ankle pain. The patient was otherwise healthy and had a body mass index (BMI) of 24.4 kg/m^2^. He denied a prior history of injury to the right foot or ankle. He was focally tender to palpation over the medial aspect of the talonavicular joint with minimal edema. Passive manipulation of the talonavicular joint elicited mild discomfort. The remaining portions of his foot and ankle were non-tender and he was neurovascularly intact. Imaging with plain radiographs was unremarkable and further imaging with MRI was obtained which aided in the diagnosis of osteoid osteoma of the talus (Figure 1).

The patient failed conservative management, which consisted of immobilization in a walking boot and oral NSAIDs. Therefore, he was scheduled for an image-guided RFA. After the ablation, his symptoms resolved within 24-48 hours after the procedure. He returned to full activity and reported no limitations. Overall, he was very satisfied with his outcome and was doing well six months post-procedure.

Case two

A 21-year-old female presented with a four-year history of pain along the medial aspect of her right hindfoot. There was no history or trauma or inciting event. She had seen several specialists prior to presenting to our institution and had been diagnosed with a stress reaction within the talus. Physical therapy improved the mobility and strength of her ankle but did not alleviate her pain. She took oral NSAIDs before bedtime, which gave her excellent relief and allowed her to sleep. She presented to our institution for an additional opinion.

**Figure 2:**
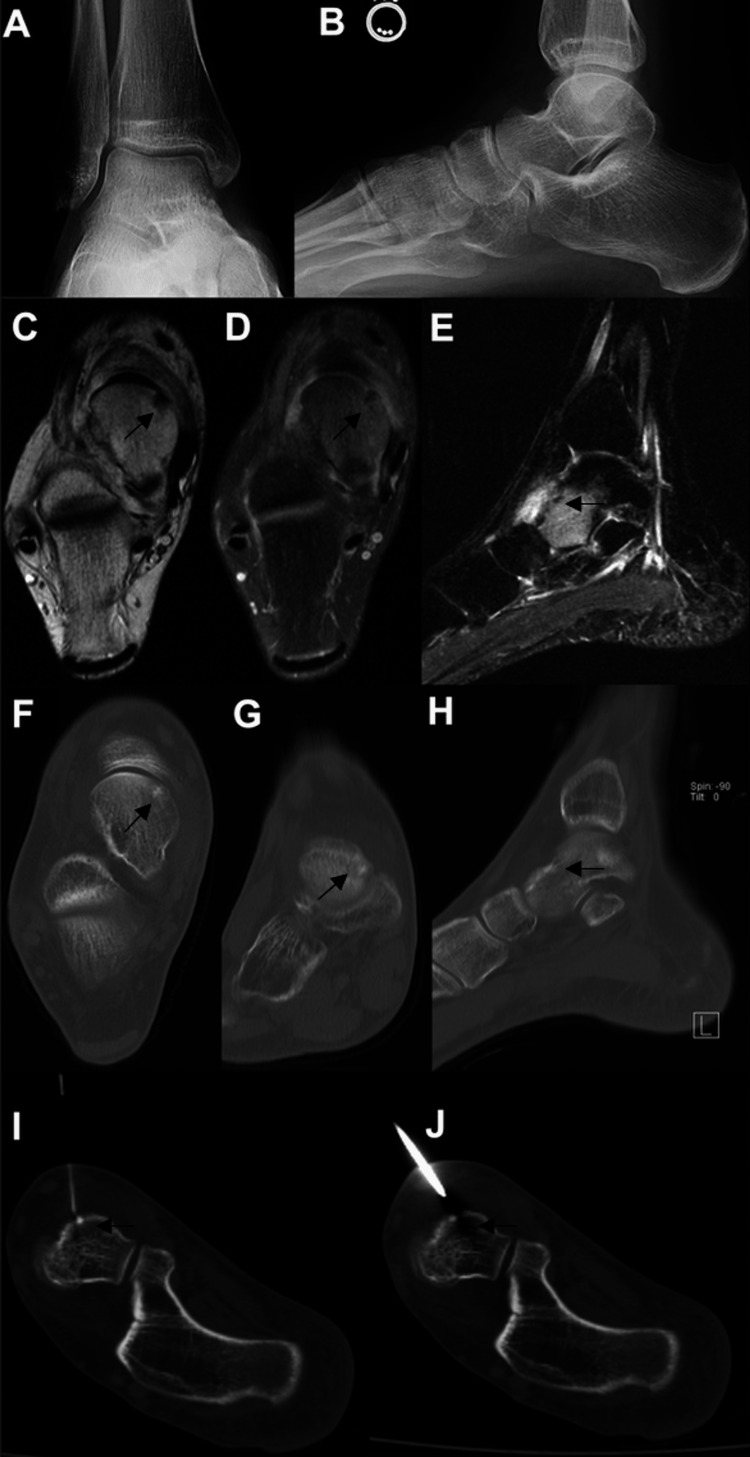
Images of the patient discussed in case two. Initial AP (A) and lateral (B) weight-bearing radiographs of the patient in case two were normal. The osteoid osteoma was identified on MRI and is highlighted by the lesion shown on the axial T1 (C) and fluid sensitive (D) sequences; extensive edema around the lesion was seen on the sagittal inversion recovery sequence (E). The nidus of the osteoid osteoma is seen on the CT axial (F), coronal (G), and sagittal (H) images. RFA localization of the lesion via CT is shown in I and J. MRI: magnetic resonance imaging; CT: computed tomography; RFA: radio-frequency ablation

The patient was otherwise healthy and had a body mass index of 20.4 kg/m^2^. Upon examination, her skin was intact, and she was focally tender about the medial aspect of her talus. Her ankle and hindfoot were flexible, but hindfoot inversion elicited mild pain in the medial aspect of her hindfoot. She had full strength, normal sensation, and palpable pedal pulses in the lower extremity. Since the patient had persistent hindfoot symptoms, an MRI was also performed at our institution, and she was diagnosed with an osteoid osteoma of the talus (Figure 2).

The patient failed conservative management, which consisted of immobilization in a walking boot and oral NSAIDs. Therefore, she was scheduled for image-guided RFA (Figure 2). Post-procedure, she was placed into a walking boot and instructed to bear weight as tolerated. The day after the ablation was performed, she noted complete relief of her symptoms. She was able to perform all of her desired activities without pain and hindfoot range of motion was no longer painful for her. She reported no complications after the ablation and was extremely satisfied with her result, with no further complications at six months post-procedure.

Case three

A 20-year-old female presented with a three-month history of right hindfoot pain. Her initial symptoms occurred when she jumped off a desk and landed awkwardly on her right foot. She was seen initially by an outside health care provider and X-rays at that time were unremarkable. She completed a course of physical therapy without notable improvement. An MRI was obtained that showed some bony edema within the talus and she was placed into a walking boot. She presented to our institution for a second opinion as she was still experiencing almost exclusively nocturnal medial hindfoot pain exacerbated by side-to-side hindfoot motion. Her pain was relieved when she took oral NSAIDs.

**Figure 3:**
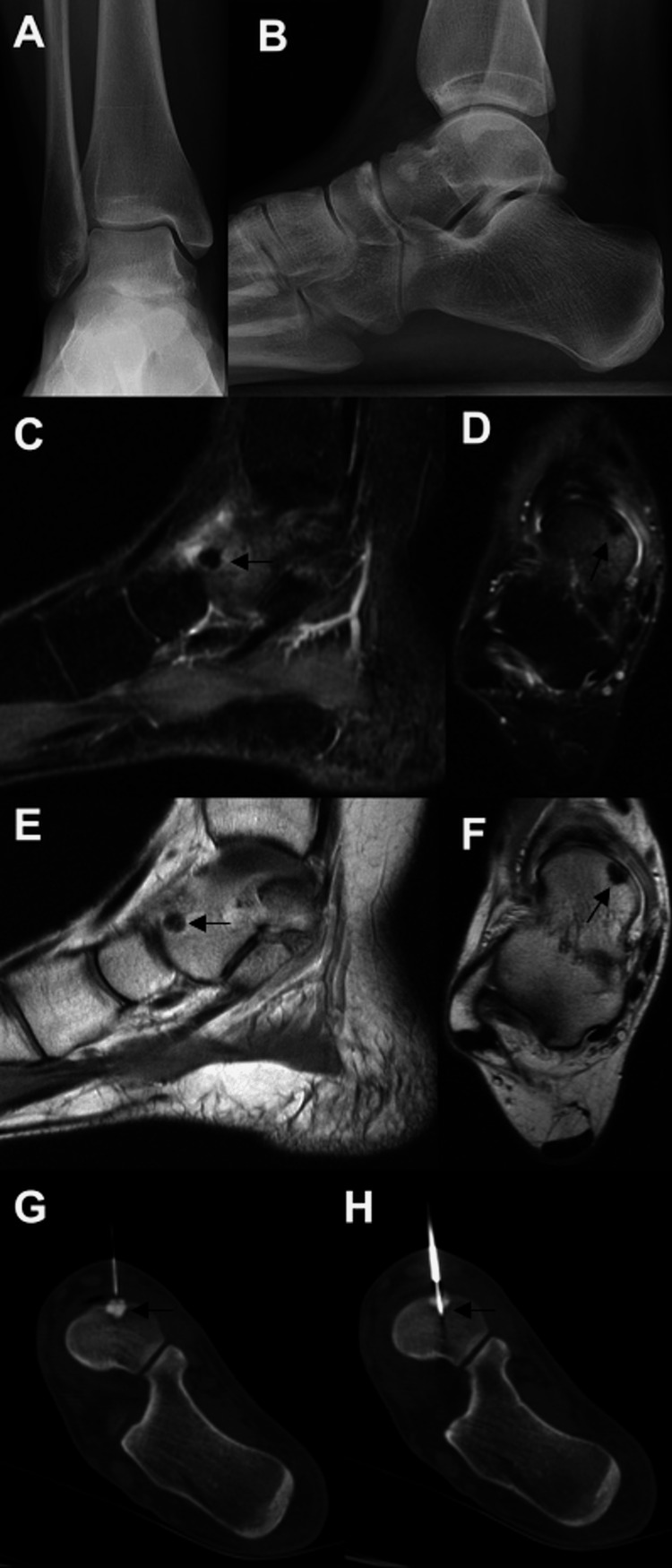
Images of the patient discussed in case three. Repeat radiographs of the ankle performed at our institution three months after initial presentation to outside health care provider (A, B) which showed a small sclerotic area in the anterior talus. MRI (sagittal (C) and axial (D) inversion recovery, and sagittal (E) and axial (F) proton density) showed a low-signal area with surrounding edema, consistent with osteoid osteoma. RFA was successfully performed on the lesion under CT guidance (G, H). MRI: magnetic resonance imaging; CT: computed tomography; RFA: radio-frequency ablation

The patient was otherwise healthy, non-smoker, with a BMI of 21.6 kg/m^2^. Upon examination, her skin was intact, and she was focally tender about the medial aspect of her talonavicular joint. Her ankle and hindfoot were flexible, but hindfoot inversion elicited mild pain in the medial aspect of her hindfoot. Her neurovascular examination was normal. Since the patient had persistent hindfoot symptoms, an MRI was repeated at our institution, and she was diagnosed with an osteoid osteoma of the dorsomedial talus (Figure 3).

The patient failed conservative management, which consisted of physical therapy, immobilization in a walking boot, and oral anti-inflammatory medications. Therefore, she underwent an image-guided biopsy and RFA of the lesion. After the ablation was performed, she reported 100% relief of her symptoms within 48 hours of the procedure. She returned to her normal activities and reported no complications after the ablation procedure.

Case four

**Figure 4:**
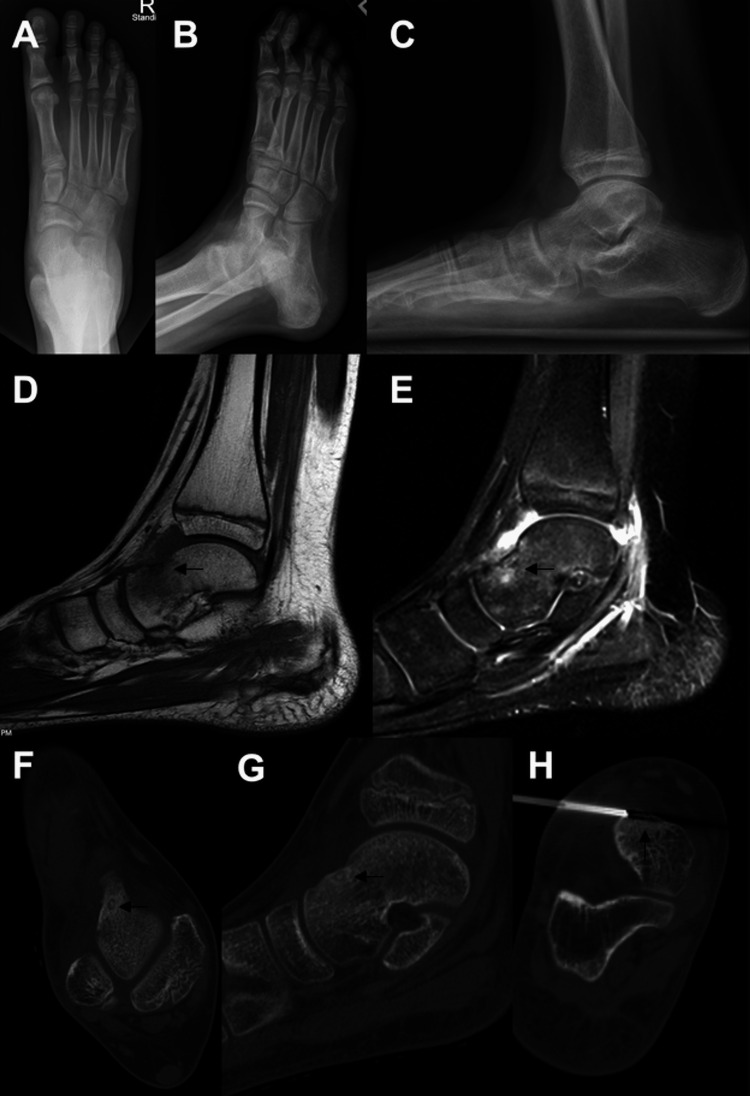
Images of the patient discussed in case four. Plain radiographs (AP (A), oblique (B), and lateral (C)) of the foot demonstrated no abnormality. Sagittal T1 MRI demonstrated a low signal area (D) with extensive edema on the fluid-sensitive sequence (E). CT confirmed the lesion with classic characteristics on axial (F) and sagittal (G) images. The patient was treated definitively with RFA (H) of the lesion under CT guidance. MRI: magnetic resonance imaging; CT: computed tomography; RFA: radio-frequency ablation

A 12-year-old otherwise healthy male presented with several months of right foot pain. The family attributed the pain to a minor injury from sports; the patient had been immobilized briefly. When he continued to have pain, an MRI was obtained, and the outside healthcare provider believed the patient had sustained a nondisplaced talus fracture. He was immobilized again and presented to our institution for a second opinion. On examination, he had mild swelling and pain over the dorsal aspect of the talus but was otherwise asymptomatic and neurovascularly intact. He did have nocturnal pain that was relieved with NSAIDs. A CT was obtained that demonstrated an osteoid osteoma in the neck of the talus (Figure 4).

The patient underwent image-guided RFA of the lesion. He was seen two weeks post-procedure and both the patient and his mother reported that he had complete improvement of his pain 24 hours after the procedure. He was able to wean out of his CAM walker boot into regular shoes. Physical therapy was initiated, and he returned to physical education and activities at four weeks post-procedure.

**Figure 5:**
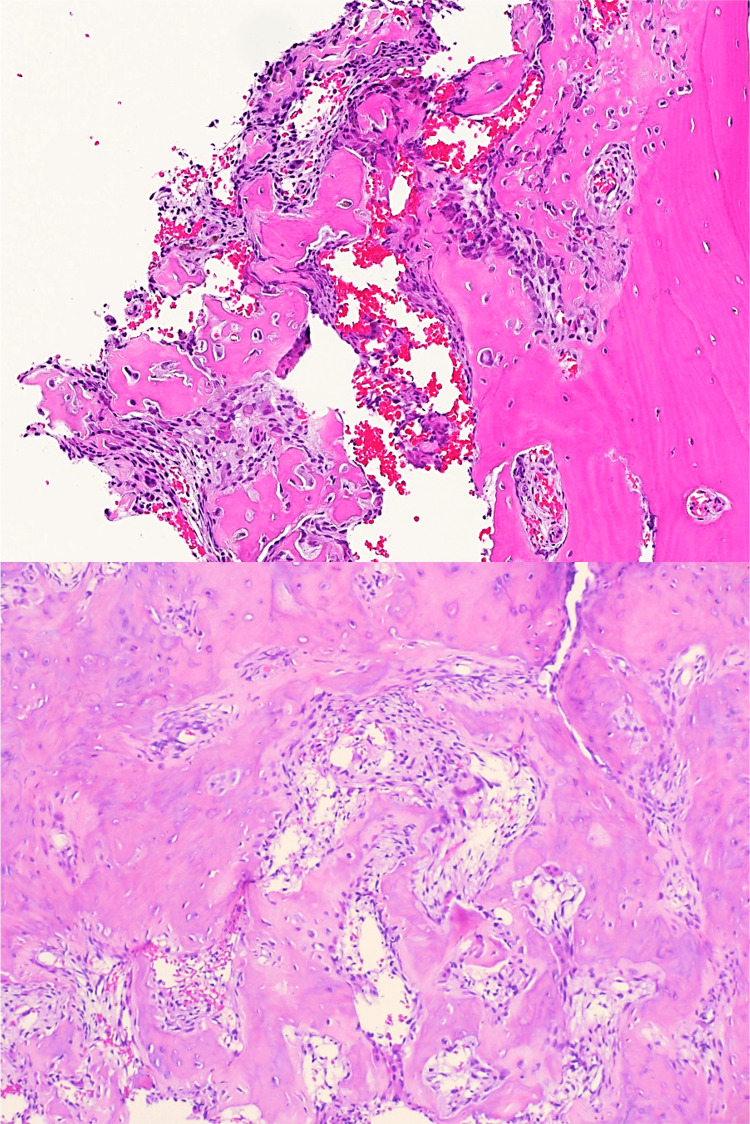
Histopathology images from one of the patients that were characteristic of osteoid osteoma. Histopathology showing cancellous bone with a bone-forming lesion, composed of interlacing trabeculae and woven bone, with a notable lining of osteoblasts and scattered osteoclast-type giant cells.

Pathology reports available for review showed bone-forming lesions composed of interlacing trabeculae and sheets of woven bone in a loose fibrovascular stroma with conspicuous osteoblastic lining and osteoclast-type giant cells, which is consistent with osteoid osteomas (Figure 5).

## Discussion

We present four cases of osteoid osteoma within the talus that presented with hindfoot pain around the talonavicular joint. NSAIDs paired with immobilization elicited inconsistent symptom reduction and all patients were subsequently treated with image-guided RFA following the failure of conservative management. Specimens were collected during the procedure and post-operative pathology showed findings consistent with the diagnosis of osteoid osteoma. All four patients achieved full relief of symptoms within 24 to 48 hours and were able to return to full activities without limitations.

Due to the often nonspecific nature of the history and physical examination findings in these patients as well as the normal plain radiographic imaging findings, further evaluation with CT or MRI is often necessary. Therefore, a high degree of clinical suspicion is important when patients present with ankle pain that does not subside with rest and lack diagnostic findings on plain radiographs. This is even true when advanced imaging like MRI is obtained, as demonstrated in the fourth case when the initial diagnosis was thought to be a fracture of the talus.

Osteoid osteomas have been previously reported within the talus by several groups [10-14]. Findings within these reports are consistent with our own, including populations of younger patients with spontaneous onset of ankle pain that is temporarily relieved with anti-inflammatories, but ultimately required intervention to remove the nidus and achieve long-term symptom relief. Of note, the need for revision surgery following failed removal of the nidus has been seen in cases following surgical removal. Methods for removal of osteoid osteomas include en bloc resection, cryo-ablation, microwave ablation, and magnetic resonance-guided focused ultrasound among others. The efficacy rates vary depending on the method used ranging from 88% for en bloc resection to 95% for percutaneous ablation, each having its own risks and benefits that should be considered when surgical intervention is indicated [15-18]. En bloc resection, the original method for removal of osteoid osteomas is associated with a lower recurrence rate than imaging-based minimally invasive surgical methods, however, they often have more complications related to longer recovery times and fractures in the bone surrounding the area [19]. Conversely, imaging-based methods allow for fewer operative complications and smaller surgical sites, however, they can have increased risks for recurrence if portions of the tumor are not eliminated [20].

## Conclusions

In our four cases of osteoid osteoma of the talus described above, we opted to perform image-guided RFA due to the intricate nature of the ankle joint and the complex surrounding neurovascular anatomy. In one of these cases, even with advanced imaging the original diagnosis was thought to be a fracture of the talus, thus demonstrating the difficult nature of identifying these lesions. These imaging-guided minimally invasive procedures resulted in complete remission of symptoms within a day of the operation with a return to normal daily activities without complications in our four cases, therefore we recommend the use of imaging-guided RFA for the management of osteoid osteomas of the talus and other anatomically complex areas of the body.
